# Hippocampal Subfield Volume in Relation to Cerebrospinal Fluid Amyloid‐ß in Early Alzheimer's Disease: Diagnostic Utility of 7T MRI

**DOI:** 10.1111/ene.70076

**Published:** 2025-02-07

**Authors:** Oluwatobi F. Adeyemi, Penny Gowland, Richard Bowtell, Olivier Mougin, Akram A. Hosseini

**Affiliations:** ^1^ Sir Peter Mansfield Imaging Centre University of Nottingha Nottingham UK; ^2^ Department of Physics University of Abuja Abuja Nigeria; ^3^ Department of Academic Neurology Nottingham University Hospitals NHS Trust, Queen's Medical Centre Nottingham UK

**Keywords:** 7 Tesla magnetic resonance imaging, Alzheimer's disease, automatic segmentation of hippocampus subfield, cerebrospinal fluid amyloid‐β, entorhinal cortex, hippocampal subfields

## Abstract

**Introduction:**

Alzheimer's disease (AD) is a neurodegenerative condition characterised by amyloid plaque accumulation and neurofibrillary tangles. Early detection is essential for effective intervention, but current diagnostic methods that enable early diagnosis in clinical practice rely on invasive or costly biomarker scanning. This study aimed to explore the utility of 7T MRI in assessing hippocampal subfield volumes and their correlation with cerebrospinal fluid (CSF) biomarkers in prodromal AD.

**Methods:**

Fifty‐six participants, including AD patients and healthy controls, underwent 7T MRI scanning. Automated segmentation delineated hippocampal subfield volumes, with subsequent normalisation to whole brain volume.

**Results:**

Significant differences in hippocampal and subfield volumes were observed in prodromal AD patients, even when they did not exhibit high MTA scores on 3T MRI or show any whole brain volume loss. Additionally, the volume of the entorhinal cortex (ERC) correlated significantly with CSF amyloid‐β levels, suggesting ERC's potential as a proxy CSF amyloid‐ß measurement. Conversely, no significant associations were found between CSF 181‐Phosphorylated‐tau or total tau levels and any hippocampal subfield volumes.

**Discussion:**

These findings show the potential use of 7T MRI, particularly in ERC assessment, as a biomarker for early AD identification. Further validation studies are warranted to confirm these results and elucidate the relationship of ERC volume with CSF biomarkers.

**Abbreviations**
AD: Alzheimer's disease
ASHS: automatic segmentation of hippocampus subfield
Aß: amyloid‐ßeta
ATN: amyloid, tau, neurodegeneration
CA: Cornu Ammonis
CNR: contrast‐to‐noise ratio
CSF: cerebrospinal fluid
DG: dentate gyrus
ERC: entorhinal cortex
HC: healthy control
MMSE: Mini‐Mental State Examination
MoCA: Montreal Cognitive Assessment
MRI: magnetic resonance imaging
MTA: medial temporal atrophy
PSIR: phase‐sensitive inversion recovery
SNR: signal‐to‐noise ratio
SUB: subiculum
TAIL: hippocampal tail
UDSNB3.0: Uniform Data set

## 1 Background

Alzheimer's disease (AD) is the leading cause of dementia, driven by a complex interplay of genetic, environmental and molecular factors [1, 2]. It is a progressive neurodegenerative disease that is characterised by neurofibrillary tangles and the accumulation of amyloid plaques [3], for which the main constituent is the amyloid‐ß (Aß) protein. Aß deposition typically begins in the neocortex, and later spreads into the hippocampus, amygdala and cingulate gyrus [4]. However, neurofibrillary tangles appear in the trans‐entorhinal and entorhinal cortices before invading the subiculum, Cornu Ammonis CA1, then CA2 and CA3 hippocampal subfields, and ultimately affecting the neocortex [5, 6]. Post mortem examination of brain tissue is the gold standard for the diagnosis of AD, and clinical diagnosis based on cognitive symptoms provides suboptimal sensitivity and specificity [7].

Early identification of AD is critical, particularly in the context of emerging Disease‐Modifying Treatments [8, 9, 10]. Pathological changes in Aß and tau can precede the onset of dementia by more than a decade [11, 12]. Aß measurements in the cerebrospinal fluid (CSF) correlate positively with cognitive performance scores, whereas Phosphorylated‐Tau and total Tau levels in the CSF correlate negatively with Mini‐Mental State Examination (MMSE) scores [13]. The presence of Aß with or without tau proteins, combined with radiological topography of neurodegeneration, currently provides the highest accuracy for the diagnosis of AD in patients who present with subjective cognitive impairment [14, 15, 16, 17]. Aß, tau‐pathology and neurodegeneration (ATN categories) are diagnostic markers to identify AD at the early stages [18, 19]. Aß pathology can be detected via Amyloid PET imaging, or by obtaining CSF through a diagnostic lumbar puncture to test for Aß and tau levels. While both methods are highly accurate for detecting AD, they are invasive, expensive or both. Furthermore, diagnostic CSF biomarker assessments do not provide topographic information about specific brain regions affected.

Structural MRI is widely used in clinical practice, with Medial Temporal Atrophy (MTA) scores serving as established neuroimaging biomarker for AD [20]. However, whole hippocampal atrophy detectable at 1.5T or 3T MRI represents a late marker of neurodegeneration, often when the disease is already advanced [21].

Ultrahigh field 7T MRI offers higher contrast‐to‐noise ratio (CNR) and signal‐to‐noise ratio (SNR) compared to 1.5T and 3T MRI which can be translated into significantly improved spatial resolution. 7T MRI consequently allows highly detailed visualisation of critical brain structures, such as hippocampal subfields, cortical layers and microstructural features, which undergo early pathological changes in AD [22]. In particular, the enhanced spatial resolution of 7T MRI allows the detection of subtle neurodegenerative changes, such as localised hippocampal atrophy, distinguishing early‐stage AD from healthy ageing. Moreover, 7T MRI has shown strong correlations between imaging biomarkers, such as hippocampal atrophy, and pathological hallmarks of AD, including neurofibrillary tangles and amyloid plaques [22, 23]. This precision enables tracking of disease progression and evaluation of therapeutic interventions.

While 7T MRI provides a direct measure of local neurodegeneration [24, 25], and validated pathological confirmation of hippocampal volume [26], the CSF pathological state of Aβ and tau do not provide information on focal neuronal loss. Additionally, structural imaging with 7T MRI has demonstrated reproducibility across multiple sites [27, 28] and provides reliable measurements of hippocampal subfield volumes [28, 29, 30]. This underscores its potential as a standardised tool for early diagnosis and monitoring in Alzheimer's disease research, with promising applications in clinical practice.

We have previously demonstrated that 7T MRI has the ability to measure differences in hippocampal subfield volume compared to healthy controls in patients with early AD who do not exhibit diagnostic high MTA scores on 3T MRI [31]. Further, we observed a reduction in hippocampal subfield volume in these patients with early AD, even without a loss of total brain volume, which correlated with their clinical amnestic impairment [31]. Here, we aim to utilise 7T MRI to precisely measure the volume of each hippocampal subfield in the early stages of AD and to correlate these measurements with Aβ and tau levels in CSF, as well as with levels of cognitive decline. Our study investigates whether 7T MRI can enhance diagnostic accuracy for AD in its early stages, as defined by the ATN criteria, before hippocampal atrophy is evident on clinical MRI.

We hypothesise that lower subfield volume will correspond to more abnormal levels of amyloid/tau status in the CSF and/or worse performance on the cognitive assessments.

## 2 Methods

### 2.1 Participants

Fifty‐six participants (31 AD patients and 25 healthy participants), aged between 40 and 79 years, were enrolled through prospective recruitment. The participants with AD fulfilled the A+T+ or A+T− criteria within the ATN framework for the AD diagnosis, confirmed by the presence of Aß, with or without tau, in the CSF of symptomatic patients who had mild cognitive impairment [32]. Participants with clear evidence of hippocampal atrophy (i.e., MTA scale > 2) on their clinical MRI at 1.5T or 3T were considered to have advanced neurodegeneration and were excluded from the 7T MRI study, as this level of atrophy typically reflects advanced neurodegeneration associated with later stages of AD [33]. This exclusion criterion was applied to focus on early‐stage cases, which are the primary target for studies of early diagnosis and intervention. Additionally, limiting the samples to participants without advanced atrophy reduces variability, enabling a clearer assessment of whether 7T MRI provides added diagnostic value over 1.5T and 3T MRI in cases where MTA atrophy is not yet diagnostic of AD.

The CSF Aß/tau measurement cut‐off values were based on the reference values (Aß_42_ range 627–1322 pg/mL; total tau range 146–595 pg/mL; and Thr181‐phosphorylated‐tau range below 68 pg/mL) as previously reported [34]. The time between CSF sampling and 7T MRI was less than 3 months. Three individuals with scan intervals ranging from 23 to 51 months were excluded from further analysis due to the substantial gap between measurements.

Inclusion criteria for the healthy control group included cognitive performance within 1.5 SD of normal in all cognitive tests [35]. As described previously, participants were assessed cognitively using the Uniform Data set (UDSNB3.0) which includes the MoCA (Montreal Cognitive Assessment) test [31]. The exclusion criteria included contraindications to Ultrahigh Field MRI, dementia (defined as loss of at least one functional ability according to the DSM‐5 Criteria [36]) and incompetency to consent.

Ethical approval for this study was obtained from a Research Ethics Committee, and all the participants provided signed informed consent (www.ClinicalTrials.gov ID NCT04992975).

### 2.2 Imaging Protocol

The participants were scanned on a Philips Achieva 7T system using a Nova Medical (Wilmington, MA, USA) single‐channel transmit, 32‐channel receive (1Tx32Rx) head coil at the Sir Peter Mansfield Imaging Centre at the University of Nottingham.

A whole‐head, T_1_‐weighted PSIR dataset was acquired first with inversion times of 725/2150 ms; TE = 3.1 ms; TR = 6.9 ms; field of view (FOV) 192 × 183 × 157 mm^3^; voxel size 0.55 × 0.55 × 0.55 mm^3^; receiver bandwidth of 300 Hz; and total scan time 12 min 25 s.

T_2_‐weighted images spanning the hippocampus were acquired using a TSE sequence: TE = 117 ms; TR = 5900 ms; FOV 224 × 224 × 64 mm^3^; voxel size 0.38 × 0.39 × 1.5 mm^3^; receiver bandwidth 155 Hz; and total scan time 04 min 43 s. The slice orientation was chosen so that the slices ran orthogonal to the longest axis of the hippocampus.

### 2.3 Image Processing and Analysis

**FIGURE 1:**
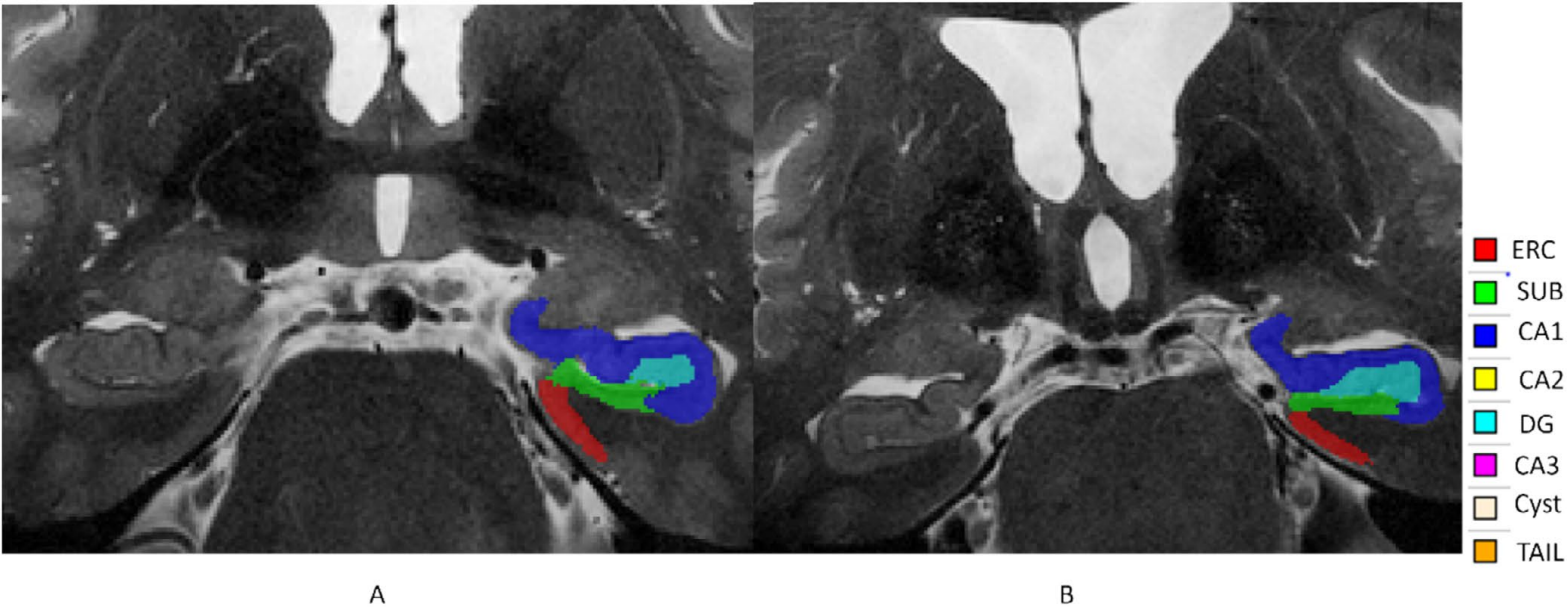
Segmentation of the hippocampus overlaid on the T_2_‐weighted image for (A) HC and (B) AD participants in the study.

Automatic Segmentation of Hippocampal Subfield (ASHS) was used to segment the hippocampus from the PSIR and T_2_‐weighted images [37] using an atlas created by the UMC Utrecht group (ashs_atlas_umcutrecht_7t_20170810) and provided by the NeuroImaging Tools and Resources Collaboratory (NITRC) [30]. In this atlas, as shown in Figure 1, the hippocampus is segmented into eight subfield ROIs: the Cornu Ammonis (CA) subfields‐ CA1, CA2 and CA3; the hippocampal tail (TAIL); the dentate gyrus (DG); the subiculum (SUB); and the cyst and the entorhinal cortex (ERC). The cyst volume was omitted because it was either very small or undetectable in all participants.

### 2.4 Percentage Volume of the Hippocampal Subfields

The volume of the whole brain differs between participants (with significant differences between male and female individuals), but we are interested in specific atrophy of the hippocampus. Therefore, we normalised the volume of the hippocampus relative to the volume of the whole brain. The whole brain volume (including the CSF and cerebellum) was extracted from the PSIR image data.

### 2.5 Statistical Analysis

Statistical analysis was carried out using a t‐test and regression analysis for comparison between AD and age‐matched healthy participants. The volumes of the left and right hippocampal subfields were combined to provide increased sensitivity. We compared the volumes of the right and left hippocampus across different subfields and the whole hippocampus in both HC and AD groups. Our results revealed no significant difference between the two hemispheres for the entorhinal cortex (ERC), Cornu Ammonis regions 1–3 (CA1‐3), dentate gyrus (DG) and the whole hippocampus, with *p*‐values ranging from 0.194 to 0.99. However, a significant difference was observed in the hippocampal tail, with a *p*‐value of 0.001.

The statistical differences in subfield volumes (CA1, CA2, CA3, ERC, TAIL, DG and SUB) between the AD and HC groups were evaluated using SPSS (version 27) and adjusted for multiple comparisons using the Bonferroni correction across the seven hippocampal subfields analysed in this study. Correlation analyses between CSF measures and subfield volumes were also adjusted using to Bonferroni correction to account for multiple comparisons.

## 3 Results

In addition to excluding three participants due to the extended interval between CSF measurement and MRI, data from three further participants with AD were excluded: one due to claustrophobia preventing imaging, one due to image degradation related to motion artefacts and one due to clinical images revealing an MTA scale of 3. Hence, the analysis was performed on data from 25 patients with AD and 25 age‐matched control participants. The participant demographics are shown in Table 1. There was no significant difference in risk factors such as smoking and alcohol habits between the AD and control participants.

**TABLE 1:** Demographics of the participants with Alzheimer's disease (AD) and healthy controls (HC).

	AD group	HC group	*p*
Number	25	25	
Age (mean, range)	60 (42–76)	62 (52–79)	0.436
Sex (male, female)	33%, 67%	44%, 56%	0.643
Mean MoCA score (mean, range)	12 (4–22)	27 (23–29)	< 0.001
Smoker	5 (19%)	9 (39%)	0.532
Alcohol (only mild to moderate consumption)	12 (44%)	12 (52%)	0.41
Time between CSF sampling and MRI (AD)	1–51 months		

Visual examination was employed to assess the accuracy of automated hippocampal segmentation. When segmentation quality was compromised by motion artefacts during scanning, participants were offered a repeat scan (*N* = 3). However, one participant's image data showed excessive motion artefacts in both the initial and repeat scans, leading to their exclusion from the study, as previously noted.

**FIGURE 2:**
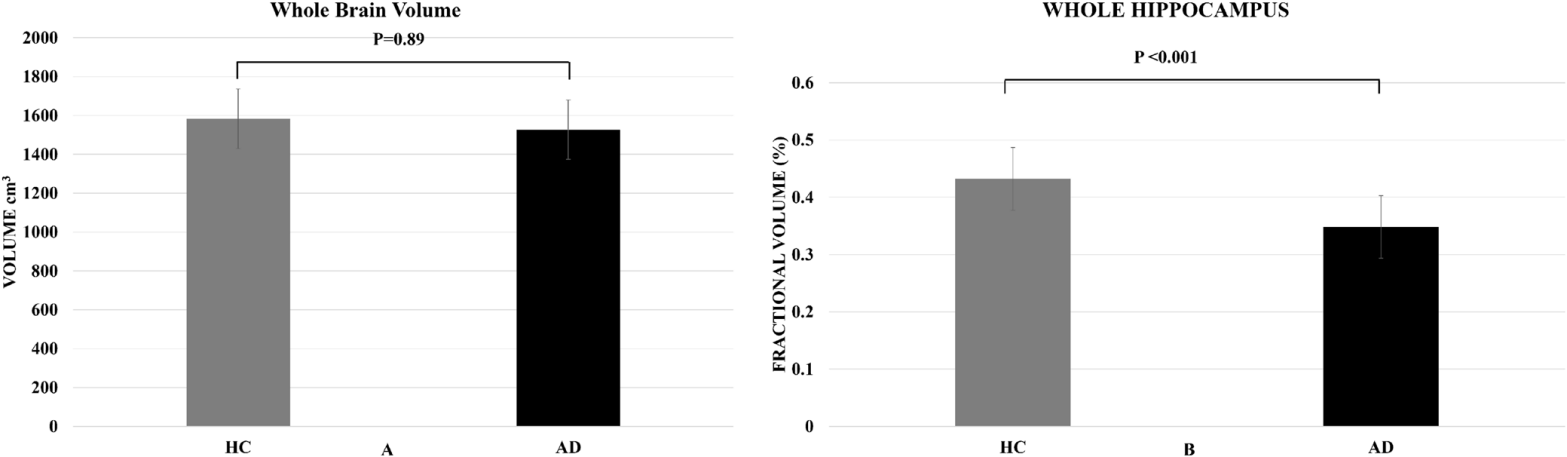
(A) The absolute volume of the whole brain averaged over the Alzheimer's disease and healthy control participant groups. There was no statistical significant between the groups. (B) The fractional volume of the whole hippocampus averaged over the Alzheimer's disease and healthy control participant groups. There was a statistically significant reduction in hippocampal volume in the AD patients compared to the controls. Error bars indicate intersubject standard deviation.

Figure 2A shows that total brain volume was smaller in the AD group compared to the healthy controls, although this difference was not significant. Figure 2B shows a significant reduction in whole hippocampal volume in patients with AD (*p* < 0.001).

**FIGURE 3:**
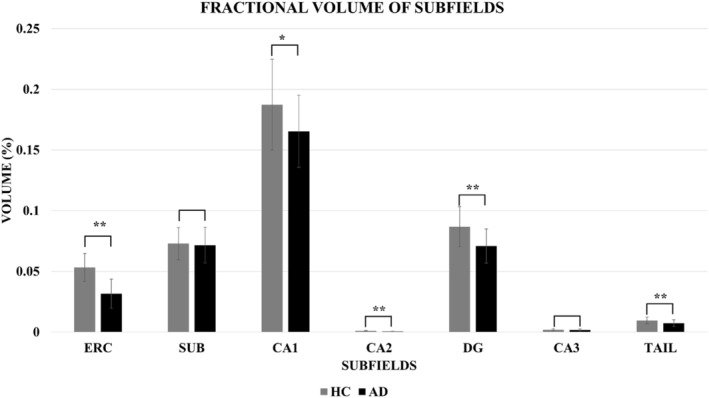
The volume of the hippocampal subfields as a percentage of whole brain volume averaged over the Alzheimer's disease and healthy control participants. Error bars indicate intersubject standard deviation; *indicates significant *p*‐value and **indicate significance with Bonferroni correction.

Figure 3 illustrates a significant reduction in the fractional volume of the ERC (*p* < 0.001), DG (*p* < 0.001), CA1 (*p* = 0.016), CA2 (*p* < 0.001) and Tail (*p* = 0.003) whereas SUB and the CA3 subfield did not attain statistical significance (*p* = 0.516 and 0.235, respectively) in patients with AD compared to controls. These findings are consistent with our previous observations reported in [31]. However, in this study, we have included a larger sample size including seven additional HC and one more AD participant. Detailed comparisons and the expanded dataset can be found in Table A2.

**FIGURE 4:**
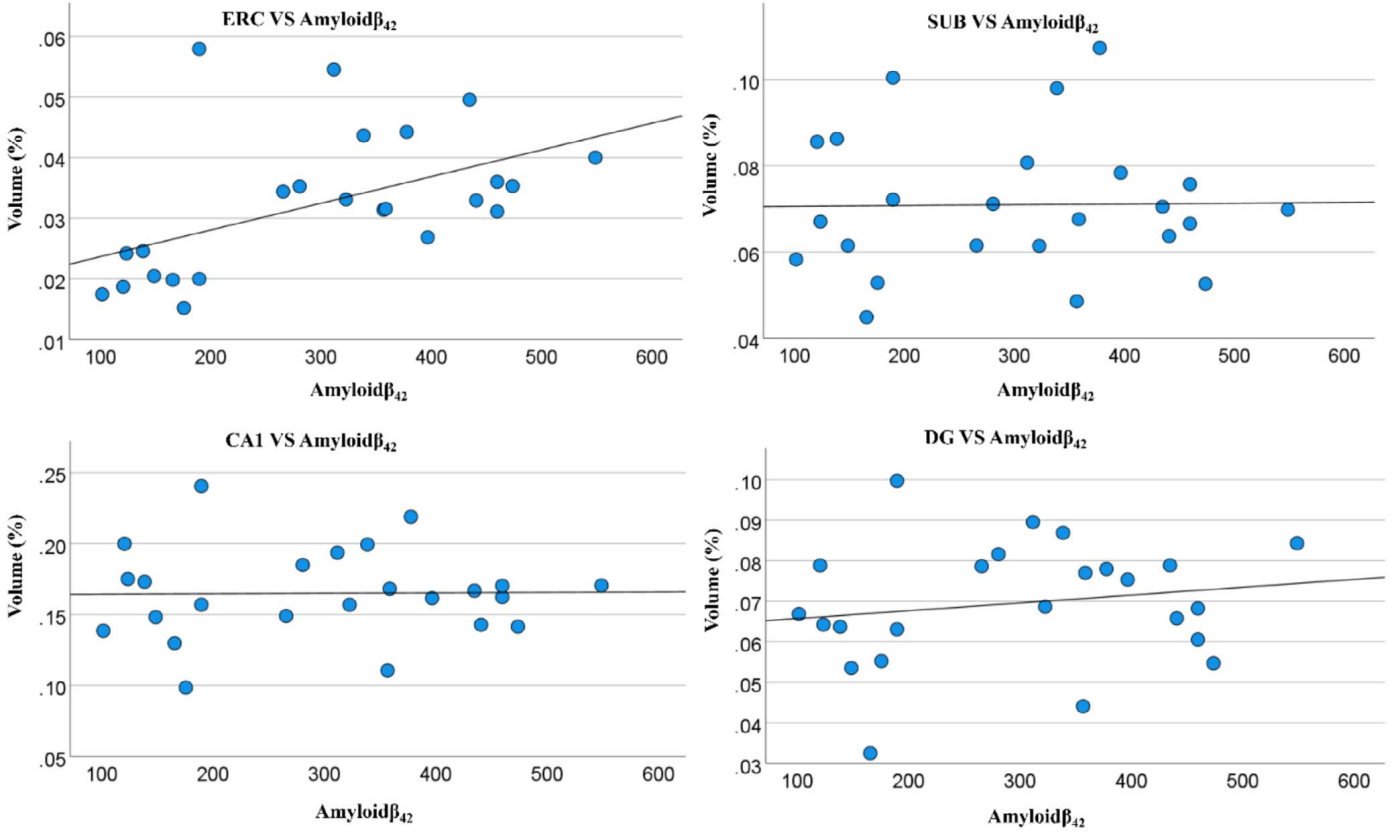
Relationship between the fractional volume of some of the hippocampal subfields for AD participants and the value of CSF amyloid‐β_42_, with linear trend lines included. CA1, Cornu Ammonis region 1; DG, dentate gyrus; ERC, entorhinal cortex; SUB, subiculum.

Figure 4 and Table 2 show the relationship between the fractional volumes of the hippocampal subfields and CSF Aβ_42_ (which was measured only in AD subjects). All subfields showed a positive correlation between fractional volume and levels of Aβ_42_ in the CSF (i.e., smaller fractional volumes were associated withlower Aβ_42_levels). However,statistical significance was only reached for the ERC (*R* = 0.596; *p* = 0.002). This corelation remained significant after Bonferroni correction. Lower ERC volume was also significantly associated with impaired cued memory in the MoCA test. Details of the cognitive assessments in relation to hippocampal subfield volumes are shown in the Table A1.

**TABLE 2:** This shows the Spearman's rho correlation analysis for associations between hippocampal subfield volumes and abnormal CSF proteins in AD participants.

Correlations				
Subfields		Amyloid‐β_42_	181‐Phosphorylated‐tau	Total tau
ERC	Correlation coefficient	0.596**	0.029	−0.144
	*p*‐value	0.002	0.902	0.533
SUB	Correlation coefficient	0.029	0.110	−0.070
	*p*‐value	0.894	0.634	0.763
CA1	Correlation coefficient	−0.001	−0.034	−0.258
	*p*‐value	0.995	0.884	0.258
CA2	Correlation coefficient	0.014	0.518*	0.358
	*p*‐value	0.950	0.023	0.132
DG	Correlation coefficient	0.136	0.113	−0.052
	*p*‐value	0.526	0.626	0.823
CA3	Correlation coefficient	0.137	0.039	−0.169
	*p*‐value	0.523	0.867	0.464
TAIL	Correlation coefficient	−0.055	−0.131	−0.312
	*p*‐value	0.799	0.571	0.169

Abbreviations: CA1‐3, Cornu Ammonis regions 1–3; DG, dentate gyrus; ERC, entorhinal cortex; SUB, subiculum.

* Indicates significant *p*‐value.

** Indicate significance with Bonferroni correction.

After Bonferroni correction, no significant associations were observed between the fractional volumes of the hippocampal subfields and the levels of CSF total tau or CSF 181‐Phosphorylated tau, as detailed in Table 2. An initially significant correlation found for Phospho‐tau in CA2 was no longer significant following the multiple correction analysis.

## 4 Discussion

In this study, we utilised ultrahigh field, 7T PSIR and T_2_‐weighted images to measure the volume of the hippocampus and its subfields in patients diagnosed with biological amyloid‐status Alzheimer's disease. These patients were categorised according to the ATN classifications as A+T−N− or A+T+N− [32], but they did not meet the AD diagnostic criteria for medial temporal atrophy (i.e., MTA scale 2 or above) on their clinical MRI data acquired at 3T. We compared the measured volumes to the levels of Aβ_42_ and tau in the CSF in patients with AD during the prodromal phase, when standard clinical MRI was not diagnostic.

In prodromal AD, we observed a significant decrease in the fractional volume of the whole hippocampus, as well as fractional reductions in the volumes of the ERC, DG, CA1, CA2 and Tail subfields (*p* < 0.001) compared to age‐matched controls [31].

Furthermore, in participants with prodromal AD, the volume of the ERC subfield (as a fraction of total brain volume) was positively correlated with the measured value of Aβ_42_ in the CSF (*R* ^2^ = 0.596 and *p* = 0.002), and this correlation remained significant after Bonferroni correction. This correlation reflects the linear relationship between abnormal CSF Aβ_42_ and ERC volume loss. These findings suggest that 7T MRI might serve as a proxy measure of CSF Aβ_42_, potentially offering an additional or alternative non‐invasive biomarker with diagnostic and monitoring value for AD.

Previous neuroimaging studies using standard MRI at 3T and 1.5T have consistently reported absolute hippocampal volume loss in AD [37, 38, 39]. In this study, we examined patients with prodromal AD who did not have a significant reduction in total brain volume, compared to age‐matched healthy controls (*p* = 0.89; Figure 2B). Despite this, using 7T MRI, we were able to identify hippocampal volume loss in these patients. Figure 2A,B demonstrates that hippocampal atrophy is not merely a secondary effect of global brain atrophy due to aging or general neurodegeneration, but rather reflects selective structural changes occurring within the hippocampus itself. This distinction is significant, as it shows the selective vulnerability of the hippocampus in AD, a region critical for memory formation, spatial navigation and cognitive processes.

The localised atrophy of the hippocampus volume indicates that the neurodegenerative changes characteristic of AD are not uniform across the brain. Rather, they exhibit a preference for specific regions, particularly those associated with essential cognitive functions. This targeted degeneration aligns with the clinical features of the disease, such as progressive memory loss and cognitive decline [40, 41]. Moreover, the observation of localised hippocampal volume loss supports the hypothesis that AD disrupts specific neural networks and circuits vital for memory consolidation and retrieval, rather than resulting from global neuronal loss [42, 43, 44].

These insights deepen our understanding of AD pathology, emphasising the importance of focusing on specific brain regions when investigating the mechanisms underlying the disease. They also suggest potential avenues for targeted interventions aimed at preserving hippocampal structure and function as a strategy to mitigate cognitive decline.

It has been previously reported that the level of CSF Aβ_42_ declines during the preclinical stage of AD [45], and correlates with delays in memory tasks in patients with Mild Cognitive Impairment (MCI) [40, 41]. Our prior research has shown a significant association between the loss of ERC and hippocampal subfields and impaired memory tasks in patients with amyloid‐positive prodromal AD [31]. Further, we have found significant positive associations between the ERC volume and the level of CSF Aβ_42_, consistent with the notion that CSF Aβ_42_ decline may serve as a marker of volume loss. The interaction between Aβ_42_ and 181‐Phosphorylated‐tau on hippocampal atrophy [42] and ERC atrophy [43] has previously been reported. Longitudinal studies further suggest that Aβ positivity is associated with accelerated ERC atrophy in non‐demented patients with elevated 181‐Phosphorylated‐tau [43]. In our study, six participants were classified as A+T− (i.e., with evidence of abnormal level of Aβ_42_, but normal level of total tau and 181‐Phosphorylated‐tau in the CSF), whereas the remainder of participants were classified as A+T+ (i.e., abnormal Aβ_42_ and tau in the CSF). We investigated the correlation between the volume of the ERC and Aβ_42_ levels in the participants with A+T− and found no significant correlation in these six AD participants. While this finding could be related to a small sample size, there is growing evidence in the literature suggesting that patients with abnormal Aβ but normal tau levels in the CSF may represent a biologically distinct subgroup within AD [44, 46]. These individuals may represent an earlier stage in the disease continuum or a subgroup less prone to tau pathology, aligning with recent findings that tau pathology tends to follow amyloid accumulation temporally [32, 47].

The ERC is known to play a crucial role in memory, spatial navigation and perception of time [48, 49]. This region is also one of the first to exhibit histopathological changes in AD before the disease spreads to the hippocampus and other brain regions [50]. The lack of correlation between ERC volume and Aβ_42_ levels in A+T− participants raises intriguing questions about amyloid‐related damage in the absence of tau pathology. It is possible that amyloid deposition alone is insufficient to drive significant neurodegeneration in the ERC without the co‐occurrence of tau pathology. This hypothesis is consistent with studies highlighting synergistic interactions between amyloid and tau in driving neurodegeneration [51, 52, 53]. Alternatively, these findings could reflect compensatory mechanisms in the ERC that initially resist amyloid‐driven structural changes. This phenomenon has been reported in preclinical AD stages or early stages of symptomatic AD, where amyloid pathology is present, but overt neurodegeneration has not yet occurred [54, 55, 56].

For the hippocampal subfields, the lack of significant correlation with CSF total tau or CSF 181‐phosphorylated tau observed in our study suggests a more complex and potentially indirect relationship between tau biomarkers and hippocampal volume loss. Tau pathology, as measured in the CSF, is thought to reflect global cortical tau burden rather than localised changes within specific hippocampal subfields, particularly during the early stages of AD progression [53, 56, 57]. Our findings align with recent studies suggesting that hippocampal volume loss may be independent of tau‐driven mechanisms, and reflect a combination of neuronal loss, amyloid‐induced synaptic dysfunction and other pathophysiological processes, including neuroinflammation and vascular factors [57, 58]. This complexity underscores the need to consider other contributors to hippocampal volume loss beyond tau accumulation alone. The ATN framework further supports the notion of distinct but interrelated biomarkers for AD [55]. In early‐stage AD, where our cohort is primarily situated, amyloid accumulation is thought to precede tau pathology temporally [57]. This temporal dissociation may explain why hippocampal volume loss correlates more closely with CSF Aβ_42_ levels than with CSF tau levels in our study. It also emphasises the importance of studying longitudinal trajectories of these biomarkers to better understand their interrelationships over time.

Finally, methodological factors could also contribute to our findings. In our study, we assessed CSF 181‐phosphorylated tau, a widely used marker for tau pathology in AD. However, emerging evidence suggests that other tau isoforms, such as 217‐phosphorylated tau and 231‐phosphorylated tau, may be more sensitive indicators of tau‐related neurodegeneration, particularly in earlier disease stages [59, 60]. Future studies employing these advanced biomarkers may reveal stronger associations with hippocampal subfield volume loss. Furthermore, combining multimodal imaging techniques, such as tau‐PET and Ultrahigh Field MRI, could provide a more nuanced understanding of regional vulnerability to tau pathology and its interplay with other biomarkers.

## 5 Conclusion

This study demonstrates the utility of Ultrahigh Field 7T MRI in detecting early structural changes in the hippocampus and its subfields in prodromal AD. Our findings reveal a positive association between ERC volume and CSF Aβ_42_, which highlights the potential of using 7T MRI as a potential proxy for amyloid burden, providing a dual assessment of Aβ pathology and neurodegeneration during a single imaging session. While this study highlights the selective vulnerability of the hippocampus in AD, it also raises important questions about the interplay of Aβ and tau pathologies in driving hippocampal degeneration, warranting further investigation in larger cohorts with longitudinal study designs.

Future research integrating advanced tau biomarkers and multimodal imaging, alongside pathological validation, could refine our understanding of the molecular underpinnings of hippocampal atrophy and strengthen the clinical utility of 7T MRI in early AD detection and monitoring.

## Author Contributions

**Oluwatobi F. Adeyemi:** data curation, formal analysis, investigation, methodology, project administration, validation, visualisation, writing – review and editing, writing – original draft, software, resources. **Penny Gowland:** methodology, project administration, resources, supervision, validation, visualisation, writing – review and editing. **Richard Bowtell:** methodology, project administration, supervision, resources, visualisation, validation, writing – review and editing. **Olivier Mougin:** formal analysis, supervision, validation, visualisation. **Akram A. Hosseini:** conceptualisation, funding acquisition, methodology, project administration, resources, supervision, validation, visualisation, writing – review and editing, writing – original draft.

## Disclosure

AAH has received funding for research from the National Institute of Health/NIA, USA (grant 1R56AG074467‐01) through her institute. AAH has received funding for Alzheimer's education, training and advice from Biogen, Eisai and Lilly.

## Consent

All participants involved in this study provided informed consent. Participants were informed about the nature, purpose, procedures, potential risks and benefits of the study. They were given the opportunity to ask questions and were assured that their participation was voluntary, with the option to withdraw at any time without any negative consequences. Written consent forms were obtained from all participants prior to their involvement in the study.

## Conflicts of Interest

The authors declare no conflicts of interest.

## Data Availability

The data that support the findings of this study are available on request from the corresponding author. The data are not publicly available due to privacy or ethical restrictions.

## Appendix A

**TABLE A1:** Spearman's rho correlation coefficients for associations between hippocampal subfield volumes and cognitive scores in Alzheimer's disease (AD) participants, the *p*‐values and the *Q*‐values (*p*‐values adjusted by Benjamini–Hochberg correction).

Correlations							
		MoCA total score	Benson Figure Immediate	Memory	Language	Attention	Executive
ERC	Correlation coefficient	0.168	0.288	0.095	0.103	−0.020	0.232
	*p*‐value	0.432	0.172	0.658	0.630	0.928	0.276
	*Q*‐value	0.504	0.290	0.674	0.679	0.928	0.399
SUB	Correlation coefficient	0.347	0.342	0.342	0.363	0.240	0.335
	*p*‐value	0.096	0.102	0.102	0.081	0.258	0.110
	*Q*‐value	0.270	0.238	0.252	0.261	0.401	0.230
CA1	Correlation coefficient	0.471*	0.396	0.514*	0.483*	0.432*	0.473*
	*p*‐value	0.020	0.055	0.010	0.017	0.035	0.020
	*Q*‐value	0.121	0.194	0.143	0.178	0.133	0.137
CA2	Correlation coefficient	0.129	0.193	0.126	0.229	0.181	0.310
	*p*‐value	0.569	0.391	0.577	0.306	0.420	0.160
	*Q*‐value	0.646	0.482	0.638	0.428	0.504	0.292
DG	Correlation coefficient	0.335	0.238	0.465*	0.356	0.292	0.451*
	*p*‐value	0.110	0.262	0.022	0.088	0.166	0.027
	*Q*‐value	0.242	0.393	0.116	0.264	0.290	0.125
CA3	Correlation coefficient	0.208	0.314	0.103	0.203	0.189	0.342
	*p*‐value	0.329	0.136	0.631	0.340	0.375	0.102
	*Q*‐value	0.446	0.259	0.663	0.447	0.478	0.268
TAIL	Correlation coefficient	0.529*	0.317	0.535*	0.441*	0.256	0.481*
	*p*‐value	0.008	0.131	0.007	0.031	0.227	0.017
	*Q*‐value	0.166	0.262	0.297	0.130	0.367	0.146

* indicate significant *p*‐value.

**TABLE A2:** The results of the *t*‐test comparing the hippocampal subfield volumes between AD and healthy control (HC) participants. A Bonferroni correction was applied to account for multiple comparisons, setting the adjusted *p*‐value threshold at 0.006.

Independent samples test										
		Levene's test for equality of variances		*t*‐Test for equality of means						
		*F*	Sig.	*t*	df	Sig. (two‐tailed)	Mean difference	Std. error difference	95% confidence interval of the difference	
									Lower	Upper
ERC	Equal variances assumed	0.001	0.977	6.023	48	0.000	0.021	0.003	0.014	0.028
	Equal variances not assumed			6.023	48	0.000	0.021	0.003	0.014	0.028
SUB	Equal variances assumed	0.871	0.355	0.654	48	0.516	0.003	0.004	−0.006	0.011
	Equal variances not assumed			0.654	47	0.516	0.003	0.004	−0.006	0.011
CA1	Equal variances assumed	0.250	0.619	2.486	48	0.016	0.025	0.010	0.005	0.046
	Equal variances not assumed			2.486	46	0.017	0.025	0.010	0.005	0.046
CA2	Equal variances assumed	0.079	0.780	4.643	46	0.000	0.001	0.000	0.000	0.001
	Equal variances not assumed			4.650	46	0.000	0.001	0.000	0.000	0.001
DG	Equal variances assumed	0.600	0.442	3.891	48	0.000	0.019	0.005	0.009	0.029
	Equal variances not assumed			3.891	46	0.000	0.019	0.005	0.009	0.029
CA3	Equal variances assumed	3.775	0.058	1.203	48	0.235	0.000	0.000	0.000	0.001
	Equal variances not assumed			1.203	45	0.235	0.000	0.000	0.000	0.001
TAIL	Equal variances assumed	0.372	0.545	3.287	48	0.002	0.003	0.001	0.001	0.005
	Equal variances not assumed			3.287	47	0.002	0.003	0.001	0.001	0.005
whole hippocampus	Equal variances assumed	0.272	0.605	4.855	48	0.000	0.089	0.018	0.052	0.126
	Equal variances not assumed			4.855	47	0.000	0.089	0.018	0.052	0.126
